# Impact of preoperative malnutrition, based on albumin level and body mass index, on operative outcomes in noncirrhosis patients with colorectal liver metastasis

**DOI:** 10.3389/fsurg.2025.1512843

**Published:** 2025-03-28

**Authors:** Yixian Guo, Yufeng Wang, Runkun Liu, Hanqi Li, Guozhi Yin, Hang Tuo, Yifeng Zhu, Yiheng Wang, Wei Yang, Zhikui Liu

**Affiliations:** Department of Hepatobiliary Surgery, The First Affiliated Hospital of Xi’an Jiaotong University, Xi'an, Shaanxi, China

**Keywords:** preoperative malnutrition, serum albumin level, BMI, liver metastases, outcomes

## Abstract

**Background:**

Serum albumin level and body mass index (BMI), acting as indicators of nutritional status, are commonly applied to predict surgical outcomes in cancer patients. This study aimed to evaluate the impact of preoperative serum albumin level and BMI on the operative outcomes of noncirrhotic patients with colorectal cancer liver metastasis who underwent hepatectomy.

**Methods:**

This was a retrospective study of medical records from the period between January 2013 and December 2022. Preoperative malnutrition was defined as hypoalbuminemia with a serum albumin level of <35 g/L before surgery or a BMI of <18.5 kg/m^2^ within 30 days before surgery. Multiple statistical methods were applied to analyze the data, including the two-independent sample *t*-test, analysis of variance, Chi-squared test, and multivariate analysis.

**Results:**

Among the 159 eligible patients, 42 (26.4%) were classified into the preoperative malnutrition group. The incidence of blood transfusion (45.24% vs. 18.80%, *P* = 0.040) was significantly higher in the malnutrition group. The drainage volume was significantly higher on the first day [65 (115) vs. 60 (80), *P* < 0.05] and the second day [50 (95) vs. 40 (79) *P* < 0.05] in the malnutrition group than that in the nonmalnutrition group. Postoperative hemoglobin levels were significantly lower in the malnutrition group (101.20 ± 2.43 vs. 108.76 ± 1.61, *P* = 0.015). Therefore, the incidence of grade Ⅱ or Ⅲ/Ⅳ complications was significantly higher in the malnutrition group (16.67% vs. 5.31% or 11.9% vs. 3.42%, *P* = 0.001), and the length of hospital stay was significantly extended [18 (12) vs. 15 (8), *P* = 0.002]. In the multivariate analysis, preoperative malnutrition [odds ratio (OR) = 5.548, 95% CI 1.508–20.413, *p* = 0.010] and operation time (OR = 1.009, 95% CI 1.002–1.016, *P* = 0.0011) were identified as independent predictors of postoperative complications.

**Conclusion:**

Preoperative malnutrition in patients who underwent hepatectomy for colorectal cancer liver metastasis was associated with worse surgical outcomes, especially aggrandizing the emergence of postoperative complications.

## 1 Introduction

Liver cancer includes primary liver cancer and metastatic liver cancer. One of the most common types of metastatic liver cancer is colorectal liver metastasis (CRLM), which occurs when cancer cells originating from the colorectal rectum migrate through the bloodstream to the liver. The liver plays a critical role in nutrient metabolism, and liver diseases can lead to an imbalance in energy metabolism (1). Protein–energy malnutrition is commonly observed in patients with liver disease (2). Malnutrition is a common and intractable problem in operative hospitalized patients, with studies showing that between 30% and 50% of cancer patients are found to be malnourished or at risk of malnutrition at hospital admission (3). Gu et al. (4) have provided strong evidence that malnutrition was associated with poor postoperative outcomes, such as wound complications and infections, across joint replacement interventions. Moreover, malnutrition leads to adverse clinical outcomes, such as higher morbidity and mortality, longer hospital stays, and increased hospitalization costs (5–7).

Hepatocellular carcinoma (HCC) is the main pathological type of primary liver cancer. In China, most hepatic malignant tumors are attributed to hepatitis B and C viral infections, which contribute significantly to the global burden of cirrhosis and subsequent HCC (8). Most patients with HCC have a liver cirrhosis background, which affects liver function, particularly the production of serum albumin (9). Generally, liver cirrhotic patients suffer from protein–energy malnutrition (10). Nutritional status and dietary patterns are factors related to the risk of HCC, which also have a critical role in the prognosis of patients with HCC (11). Malnutrition is proposed to determine the postoperative outcome of surgery (12), which is a prognostic factor in patients with HCC and has been shown to negatively affect survival in these patients (13). Prognostic nutritional index and BMI are established immune-nutritional indices associated with postoperative outcomes in HCC (14). The albumin–bilirubin score may serve as a useful predictor of energy malnutrition in patients with HCC (15, 16). Serum albumin level and body mass index are generally used as indicators of malnutrition, with recent reports showing that both are correlated with postoperative mortality (17). The subjective global assessment (SGA), also known as the global clinical assessment (GCA), was proposed by Detsky in 1987 as a nutritional evaluation tool. It is currently used as a method for both screening and evaluating nutritional status, but it is frequently used as an evaluation tool (18).

Several studies have confirmed the association between preoperative malnutrition and surgical outcomes in colorectal cancer and HCC (19–21). However, the effects of malnutrition on colorectal liver metastasis (CRLM), especially in patients without cirrhosis background (22), have not been evaluated. Prehabilitation before a major operation, including muscle training and nutritional support, has been investigated comprehensively and shown to improve postoperative complications and reduce the length of hospital stay (23). However, for noncirrhotic patients with CRLM who are qualified for hepatectomy, the evidence supporting the efficacy of such adjuvant nutritional therapy for postoperative intervention is still lacking.

Therefore, this study aimed to investigate the correlation between preoperative malnutrition, based on albumin level and BMI, and operative outcomes, short-term prognosis, and complications in CRLM patients who underwent hepatectomy. Meanwhile, we prospect to provide clinical guidance for perioperative nutrition support.

## 2 Methods

### 2.1 Data acquisition and eligibility criteria

This was a retrospective study employing information from patients who underwent curative liver resection for CRLM between January 2013 and December 2022. The included patients were pathologically confirmed to have colorectal cancer. The exclusion criteria were as follows: (1) patients with liver diseases, such as hepatitis B and C and cirrhosis; (2) patients with artificially altered serum albumin levels due to medication or albumin infusion; and (3) patients lost to follow-up. The same surgical team performed all operations. The protocol of this retrospective study was approved by the institutional review board of the hospital.

### 2.2 Nutritional assessment and data collection and definition

In this study, preoperative malnutrition was defined as hypoalbuminemia with a serum albumin level of <35 g/L before surgery or a BMI of <18.5 kg/m^2^. The World Health Organization (WHO) defines BMI < 18.5 kg/m^2^ as underweight. In addition, the SGA nutritional score, Nutritional Risk Screening (NRS) 2002, and other nutritional assessment tools were used to assist in the assessment of malnutrition in malnourished patients. The SGA was used to classify patients' nutritional status into three categories, namely, normal nutrition (Grade A), mild malnutrition (Grade B), and severe malnutrition (Grade C), based on factors such as weight change, nutrient intake, gastrointestinal symptoms, edema, subcutaneous fat and muscle depletion, and functional activity. Tumor staging was performed according to the Colon Cancer, Version 2.2021, National Comprehensive Cancer Network (NCCN) Clinical Practice Guidelines in Oncology (24).

The following variables were analyzed to evaluate operative outcomes: operation time (minutes), intraoperative estimated blood loss (EBL, ml), transfusion, type of surgical method, total and postoperative length of hospital stay (days), drainage volume (ml), preoperative and postoperative hemoglobin difference (g/L), and white blood cell count (10^9^/L). To evaluate patients' performance and functional status, we used the Eastern Cooperative Oncology Group/World Health Organization Performance Status (ECOG/WHO PS) and the American Society of Anesthesiologists (ASA) score.

### 2.3 Statistics

We divided patients into the malnutrition and nonmalnutrition groups. Statistical analyses were carried out using the SPSS v20 software (Chicago, IL, USA). We compared the means of two independent samples with continuous normally distributed variables using a two-independent sample *t*-test, and a separate variance estimation *t*-test was used for data with uneven variance. Analysis of variance (ANOVA) was applied when comparing the means of multiple samples to avoid type I error. In addition, the Chi-squared test was used to compare two disorder variables, and the Bonferroni correction was applied for pairwise comparisons of multiple rates. Next, the rank-sum test was used for cases where the data sample did not conform to a normal distribution or the variance was not uniform. Finally, univariate and multivariate analyses were used. Continuous variables with nonnormal distribution were expressed as the median (interquartile range). Demographic and perioperative characteristics were summarized using descriptive analyses, and all qualitative values were presented as mean ± standard deviation unless expressed otherwise. Correlation analysis between two continuous variables was performed using Spearman's test. A *P*-value of <0.05 was considered statistically significant.

## 3 Results

### 3.1 Patient demographics

**Figure 1:**
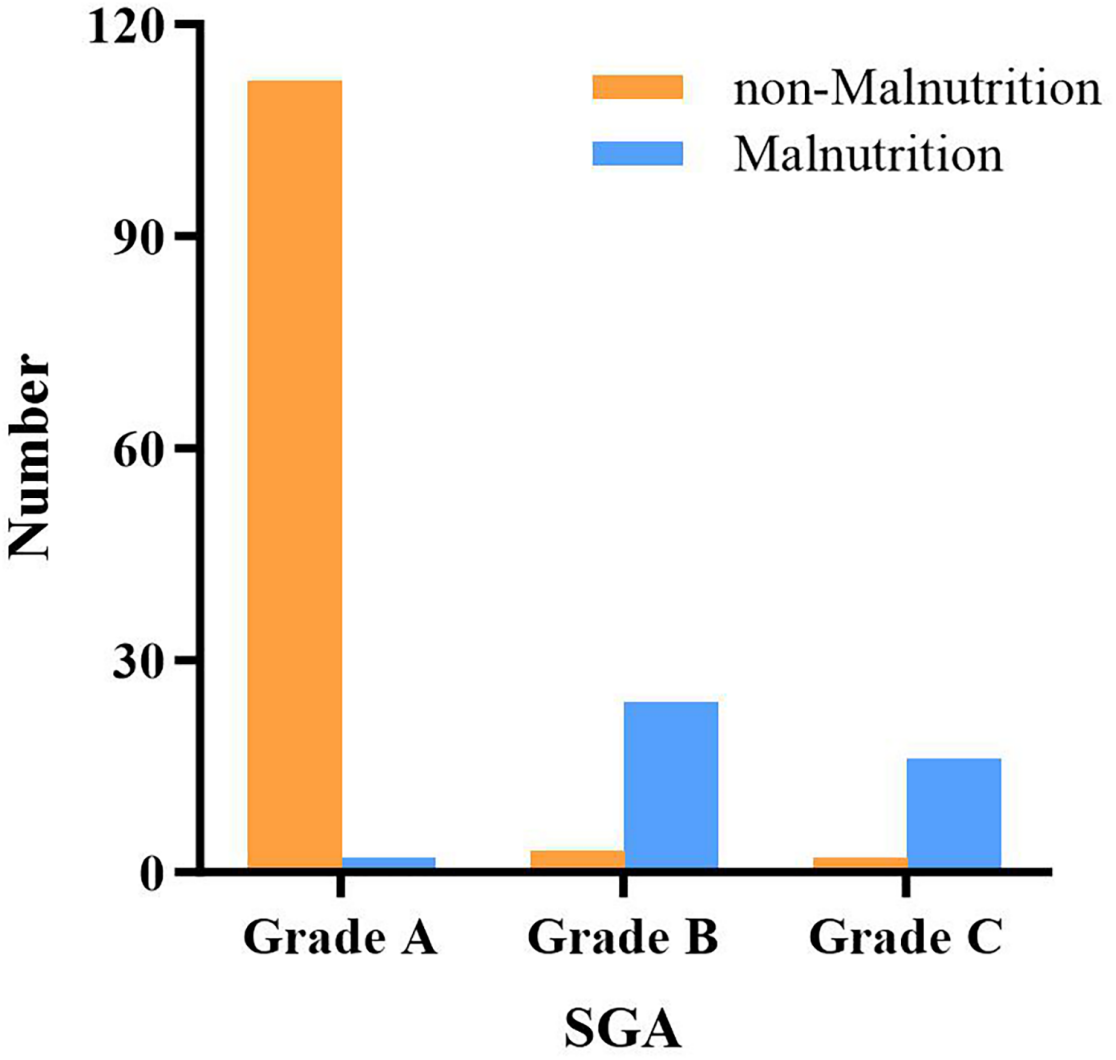
Patients were divided into malnutrition and nonmalnutrition groups according to SGA.

A total of 42 patients were identified with preoperative malnutrition, accounting for 26.4% of 159 eligible patients. The results of the SGA-aided assessment of malnutrition groups, shown in Figure 1, were consistent with the data based on serum albumin level and BMI. The baseline characteristics of the patients are presented in Table 1. There was no significant difference in gender between both groups. However, the average age of patients in the malnutrition group was higher than that in the nonmalnutrition group (62.02 ± 1.51 vs. 56.38 ± 1.01, *P* < 0.05). Moreover, the proportion of patients with severe comorbidities and limited physical activity was significantly higher in the malnutrition group (ASA Class 3, 64.29% vs. 42.74%, *P* = 0.022). The prevalence of underlying diseases, including type 2 diabetes mellitus (T2DM) (malnutrition group vs. nonmalnutrition group: 7.14% vs. 13.68%, *P* = 0.263) and hypertension (16.67% vs. 23.08%, *P* = 0. 385), was similar in both groups. In addition, CA19-9 (*P* = 0.633), CA125 (*P* = 0.052), and international normalized ratio (INR) (*P* = 0.299) levels were also similar in both groups. In contrast, compared with the nonmalnutrition group, the malnutrition group had significantly lower carcinoembryonic antigen (CEA) [2.93 (13.29) vs. 4.66 (19.59), *P* = 0.047], preoperative hemoglobin (114.85 ± 2.38 vs. 124.81 ± 1.68, *P* = 0.002), and platelet count [144 (117) vs. 175 (82.5), *P* = 0.022].

**Table 1:** Patient demographics.

**Variable**	Malnutrition		*P-*value
	No (*n* = 117, 73.58%)	Yes (*n* = 42, 26.42%)	
Age (years)	56.38 ± 1.01	62.02 ± 1.51	0.004
Sex (*n*/%)			
Male	58 (49.57%)	24 (57.14%)	0.401
Female	59 (50.43%)	18 (42.86%)	
ASA (*n*/%)			
2	67 (57.26%)	15 (35.71%)	0.022
3	50 (42.74%)	27 (64.29%)	
T2DM (*n*/%)	16 (13.68%)	3 (7.14%)	0.263
Hypertension (*n*/%)	27 (23.08%)	7 (16.67%)	0.385
CA19-9 (U/ml)	21.30 (44.58)	15.74 (70.66)	0.633
CA125 (U/ml)	11.70 (8.28)	15.80 (24.19)	0.052
CEA (U/ml)	4.66 (19.59)	2.93 (13.29)	0.047
Preoperative Hb (g/L)	124.81 ± 1.68	114.85 ± 2.38	0.002
Platelet (10^9^/L)	175 (82.5)	144 (117)	0.017
INR	1.02 (0.11)	1.04 (0.13)	0.229

ASA, American Society of Anesthesiologists; CA19-9, carbohydrate antigen 19-9; T2DM, type 2 diabetes mellitus; CA125, carbohydrate antigen 125; CEA, carcinoembryonic antigen; INR, international normalized ratio; Hb, hemoglobin.

### 3.2 Operative data

Table 2 presents the surgical data of each group. No significant association was found between surgical methods and malnutrition or in the conversion rate of laparotomy. Moreover, there was no significant difference in operation time (*P* = 0.860) and EBL (*P* = 0.940) between both groups, but transfusion (malnutrition group vs. nonmalnutrition group, 45.24% vs. 18.80, *P* = 0.040) was higher than that in the malnutrition group. The drainage on the postoperative third day and time of drainage tube removal after operation were similar between the two groups. However, the drainage volume in the malnutrition group was significantly higher on the first day [65 (115) vs. 60 (80), *P* < 0.05] and the second day [50 (95) vs. 40 (79), *P* < 0.05] than that in the nonmalnutrition group. The postoperative hemoglobin of the malnutrition group was significantly lower (101.20 ± 2.43 vs. 108.76 ± 1.61, *P* = 0.015), although there was similarity in the difference of hemoglobin between both groups before and after operation (16.00 ± 1.52 vs. 14.10 ± 2.79, *P* = 0.537), which could be seen that patients in these two groups were prone to anemia during perioperative period. Nevertheless, there was no difference in white blood cell and neutrophil percentage between both groups. In short, the incidence of grade Ⅱ or Ⅲ/Ⅳ complications was significantly higher in the malnutrition group (16.67% vs. 5.31% or 11.9% vs. 3.42%, *P* = 0.001), while the length of hospital stay [18 (12) vs. 15 (8), *P* = 0.002] was significantly extended.

**Table 2:** Operative data.

**Variable**	Malnutrition		*P-*value
	No (*n* = 117, 73.58%)	Yes (*n* = 42, 26.42%)	
Surgical method (*n*/%)			
Laparoscopy	49 (41.88%)	17 (40.48%)	0.761
Open	58 (49.57%)	23 (54.76%)	
Open conversion	10 (8.55%)	2 (4.76%)	
Operation time (min)	217.5 (75)	220 (108)	0.860
EBL (ml)	400 (400)	350 (675)	0.940
Transfusion (%)	22 (18.80%)	19 (45.24%)	0.040
Complication (*n*/%)			
Ⅱ	6 (5.13%)	7 (16.67%)	0.001
Ⅲ/Ⅳ	4 (3.42%)	5 (11.90%)	
Postoperative drainage (ml)			
First day after surgery	60 (80)	65 (115)	0.036
Second day after surgery	40 (79)	50 (95)	0.022
Third day after surgery	22 (74)	30 (45)	0.562
Drainage tube removal time (day)	6 (3)	7 (3)	0.206
Postoperative hemoglobin (g/L)	108.76 ± 1.61	101.20 ± 2.43	0.015
Hemoglobin difference (g/L)	16.00 ± 1.52	14.10 ± 2.79	0.537
White blood cell count	9.70 (5.85)	8.64 (5.23)	0.127
Neutrophil percentage (%)	84.10 (16.30)	83.10 (15.13)	0.641
Hospital stay (days)	15 (8)	18 (12)	0.002

EBL, estimated blood loss.

### 3.3 Pathology and short-term outcomes

**Figure 2:**
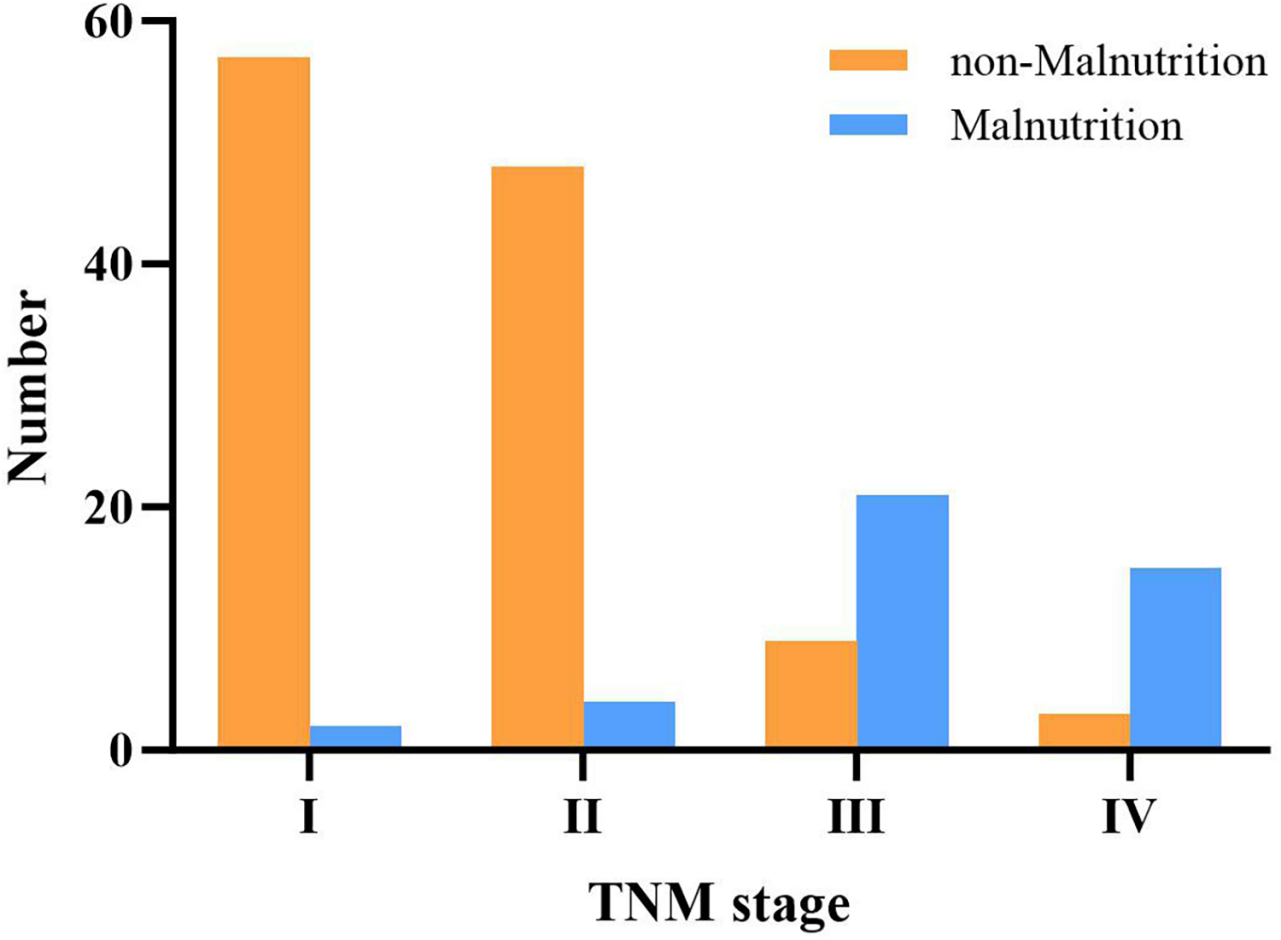
TNM stages of patients in the malnutrition and nonmalnutrition groups.

The oncological findings and short-term survival between the two groups are summarized in Table 3. The number (*P* = 0.760) and diameter (cm) [3.5 (3.75) vs. 4.0 (2), *P* = 0.848] of liver metastases were similar between the malnutrition and nonmalnutrition groups. Meanwhile, we found that mortality was not significantly different in both groups during short follow-up periods (30 days and 6 months) after surgery. Furthermore, we divided nonmalnourished and malnourished patients into four stages according to the TNM staging system and found that the more advanced the tumor stage, the higher the probability of malnutrition (Figure 2).

**Table 3:** Pathology and short-term outcomes.

**Variable**	Malnutrition		*P-*value
	No (*n* = 117, 73.58%)	Yes (*n* = 42, 26.42%)	
Number of metastases			
1	66 (56.41%)	23 (54.76%)	0.760
2	18 (15.38%)	5 (11.91%)	
≥3	33 (28.21%)	14 (33.33%)	
Tumor size (cm)	4.0 (2)	3.5 (3.75)	0.848
Mortality (*n*/%)			
Within 30 days	0 (0%)	1 (2.38%)	0.264
Within 6 months	1 (0.85%)	2 (4.76%)	0.171

### 3.4 Risk factors of postoperative complication

Among the survivors, preoperative malnutrition (*P* < 0.001), operative time (*P* < 0.001), and EBL (*P* < 0.001) were significantly associated with postoperative complications, which were observed in univariate analysis. Furthermore, as shown in multivariate logistic regression analysis, preoperative malnutrition [odds ratio (OR) = 5.548, 95% CI 1.508–20.413, *p* = 0.010], and operation time (OR = 1.009, 95% CI 1.002–1.016, *P* = 0.0011) were independent predictors of postoperative complication. See Table 4 for details.

**Table 4:** Univariate and multivariate analysis of factors associated with complication.

**Variable**		Univariate analysis		Multivariate analysis	
		Complication		OR (95%CI)	
	Yes (*n* = 22, 13.84%)	No (*n* = 137, 86.15%)	*P*-value		*P*-value
Age (years)	57.91 ± 2.00	57.87 ± 0.95	0.985		
Male (*n*/%)	12 (54.55%)	70 (51.09%)	0.950	5.548 (1.508–20.413)	0.010
Preoperative malnutrition (*n*/%)	13 (59.09%)	10 (7.30%)	<0.001		
ASA (*n*/%)					
2	8 (36.36%)	73 (53.28%)	0.094		
3	15 (68.18%)	63 (45.99%)			
CA19-9	27.31 (85.52)	17.73 (109.04)	0.476		
CA125	13.55 (21.15)	11.85 (10.23)	0.662		
CEA	3.07 (32.64)	4.05 (17.37)	0.650		
Platelet (10^9^/L)	159.5 (143.0)	170.5 (84)	0.473		
Operation time (min)	280 (160)	210 (62)	<0.001	1.009 (1.002–1.016)	0.011
EBL (ml)	1,000 (1,500)	300 (400)	<0.001	1.001 (1.000–1.002)	0.142

OR, odds ratio; 95% CI, 95% confidence interval; ASA, American Society of Anesthesiologists; CA19-9, carbohydrate antigen 19-9; CA125, carbohydrate antigen 125; CEA, carcinoembryonic antigen; EBL, estimated blood loss.

## 4 Discussion

Cancer patients with malnutrition often experience a combination of weight loss and dysmetabolism. Whether due to voluntary or involuntary caloric restriction, malnutrition increases the risk of cachexia. Unfavorably, both conditions contribute to the depletion of fat and protein storage, negatively impacting treatment tolerance, complication rates, and survival (25). There is a high prevalence of malnutrition in cancer patients (26), with 50% who are presenting for the first time being malnourished or at risk of malnutrition (27) and 80% who are malnourished during treatment (28), which seriously affects the efficacy of anticancer treatment and patient outcomes (29, 30), contributing to the huge global disease burden.

Malnutrition is a matter of particular concern in patients with primary liver cancer, which is distinguished from liver metastases due to concomitant underlying cirrhosis. The present research was designed to investigate whether preoperative malnutrition impacted operative outcomes in noncirrhotic patients with CRLM. Here, we demonstrated that preoperative malnutrition was associated with high blood transfusion, major postoperative complications, postoperative drainage of the earlier stage, postoperative hemoglobin, and longer hospital stays than those without malnutrition. Moreover, there was no significant difference in mortality rates within 6 months after surgery. The present study reported for the first time that preoperative malnutrition, based on albumin level and BMI, is associated with postoperative outcomes and short-time prognosis of the patients who underwent hepatectomy for CRLM, although the mechanisms remain largely unknown.

Here, we observed that preoperative malnutrition was closely associated with adverse surgical outcomes. First, the blood transfusion and major complications were significantly higher in the malnutrition group. We speculated that high blood transfusion and hypohemoglobin may result from conditions such as malnutrition, severe hemorrhage, and abnormal coagulation factors. In addition, hypohemoglobin before partial hepatectomy was related to the worse nutriture. Multiple studies revealed a better prognosis after curative treatment of malignant tumors if the nutritional status had been optimized before any intervention (31–34). The elder patients were prone to malnutrition. Although the preoperative platelet and INR levels of patients were within the normal range in the malnutrition group, the platelet was lower, and INR was higher than that of those in the normal groups, which is more likely to affect blood loss and blood transfusion. Secondly, we identified that malnutrition was associated with postoperative drainage of the earlier stage. It is well known that malnutrition easily leads to tissue edema, friability, ascites, and susceptibility to infection. Moreover, malabsorption frequently occurring in CRLM patients also tends to result in hypoalbuminemia, which increases the risk of drainage.

Additionally, previous researches suggest that malnutrition increases the incidence of postoperative complications in patients with surgery (35–37). Boram et al. (5) reported that malnutrition is an independent risk factor for complications of pancreaticoduodenectomy in pancreatic head cancer. Here, we performed a multivariate analysis to determine the risk factors for complications after hepatectomy for CRLM and demonstrate that preoperative malnutrition and operation time are independent risk factors for complications. However, it is still unknown why patients with malnutrition have worse postoperative outcomes. Further study was needed to elucidate the exact mechanism of these adverse effects of malnutrition.

While malnutrition is a modifiable risk factor for surgical complications, special attention should be paid to nutritional support, including proper nutritional assessment and therapy by a multidisciplinary team. Nutritional therapy should center on providing enough energy and protein to manage the increased requirements of cancer and partial hepatectomy and overcome the highly catabolic state. Nutritional assessment is of great importance in patients with CRLM, and the goal is to establish a baseline body composition assessment and continue with frequent follow-ups to monitor the response to nutritional therapy. Multiple studies reported that adequate nutritional intervention may improve clinical outcomes and reduce hospital costs in patients (38, 39). For all of this, the study has several limitations. As a retrospective study, selection bias and information bias were inevitable. Therefore, strict and clear criteria were set up for the inclusion and exclusion of research objects, which could better represent the population from which they were derived, to avoid bias. Moreover, we just chose the albumin level and BMI to assess malnutrition because other information, such as bioelectrical impedance, midarm muscle circumference, and muscle mass was not available. A more comprehensive assessment was needed. In addition, blinded methods were used to collect patient information, and objective indicators were used as research information to reduce bias. In the end, this study was performed in a single center, and we need more centers to perform further prospective studies to elucidate the relationship between malnutrition and adverse operative outcomes.

In conclusion, this study suggests that preoperative nutritional assessment and the appropriate interventions can benefit CRLM patients who suffer from colorectal cancer liver metastasis without a cirrhosis background and who underwent hepatectomy to restore energy deficit, avoid worse surgical outcomes, and improve short-term prognostic outcomes. This approach is consistent with the recommendations from the European Society for Clinical Nutrition and Metabolism (ESPEN) expert group (40).

## Data Availability

The raw data supporting the conclusions of this article will be made available by the authors, without undue reservation.
